# 

**Published:** 1856-04

**Authors:** 


### THE JOURNAL OF PUBLIC HEALTH.

### CONTENTS of No. V.

Municipal Government: its true Sphere and Duties .......... 1

Prevention and Cure .......... 8

Hygiene in France .......... 19

**EPITOME OF SANITARY LITERATURE:**

Parasitic Developments in the Human Body .......... 26
Notes on the Mining Districts .......... *ib.*
Prevention of Death by Chloroform .......... 29
The *Lancet* Sanitary Commission .......... *ib.*
Diet of Charitable Institutions .......... 30
Composition of Bread .......... 31
Salt in Agriculture .......... 32

**ORIGINAL COMMUNICATIONS:**

Coke Poisoning in a Church. By John Davy, M.D., F.R.S. .......... 33
Diseases of Colliery Operatives in South Lancashire. By W. J. Cox, Esq., M.R.C.S. .......... 39
Sanitary Reform in Relation to Social and Moral Development. By Benjamin Daniel, M.R.C.S. .......... 43
Nursery Government in its Sanitary Aspects. By T. H. Barker, M.D. .... 52
The First Food of Infants. By F. J. Brown, M.D. .......... 59
The Dictionary of Foods and Drinks: a Compendium of Alimentary Substances in use throughout the World: their Histories, Dietetic Properties, and Adulterations. Part IV. .......... 61

**SANITARY AND SOCIAL SCIENCE:**

*Reports on Cities, Towns, and Districts.*—Dudley.—Paddington.—Croydon .. 66

*Miscellanea.*—Pauperism in England and Wales.—Grain and Flour imported into Ireland.—Homes for Needlewomen.—Poisonous Effects of Turpentine Vapour.—Poisoning by Sulphuret of Carbon among workmen in India-Rubber Manufactories. — Sanitary Application of Charcoal. — Mortality in the Cities of the United States .......... 81

**PROGRESS OF EPIDEMICS in Twenty-Eight Towns and Districts** .......... 85

**SANITARY LEGISLATION:**

A complete resumé of, including Notices of Bills and Reports on Public Health, subjects issued by the Legislature during the past quarter, and the second Report of the Postmaster-General .......... 100

**TRANSACTIONS of the EPIDEMIOLOGICAL SOCIETY of London:**

A Plan for providing the Poor with Nurses in times of Epidemic Sickness. By Dr. Sieveking .......... 1

On Cider and the Vegetable Acids as preventive of Cholera. By J. H. Tucker, Esq. .......... 10

Proceedings of the Society .......... 24

